# Supplementary material for: Modeling glioblastoma heterogeneity as a dynamic network of cell states
Source: Mol Syst Biol. 2021 Sep 16;17(9):e10105. doi: 10.15252/msb.202010105 (PMC8444284; doi:10.15252/msb.202010105)
Supplement: Supplementary file 6 — Source Data for Figure 5 [file MSB-17-e10105-s004.zip › Figure5A_sourcedata/GSEA_3071/nosigdb_stateIII.GseaPreranked.1622795155562/FELDMAN_2020_LOWRNA.html]

Details for gene set FELDMAN\_2020\_LOWRNA[GSEA]

|  || Dataset | state33071 |
| Phenotype | NoPhenotypeAvailable |
| Upregulated in class | na\_pos |
| GeneSet | FELDMAN\_2020\_LOWRNA |
| Enrichment Score (ES) | 0.30096477 |
| Normalized Enrichment Score (NES) | NaN |
| Nominal p-value | NaN |
| FDR q-value | 1.0 |
| FWER p-Value | 0.0 |
Table: GSEA Results Summary

  

Fig 1: Enrichment plot: FELDMAN\_2020\_LOWRNA      
 Profile of the Running ES Score & Positions of GeneSet Members on the Rank Ordered List

  

| PROBE | GENE SYMBOL | GENE\_TITLE | RANK IN GENE LIST | RANK METRIC SCORE | RUNNING ES | CORE ENRICHMENT || 1 | RPL31 |  |  | 2 | 0.687 | 0.0037 | Yes |
| 2 | RPL38 |  |  | 5 | 0.638 | 0.0071 | Yes |
| 3 | RPLP0 |  |  | 6 | 0.633 | 0.0110 | Yes |
| 4 | RPS29 |  |  | 7 | 0.630 | 0.0149 | Yes |
| 5 | RPS12 |  |  | 8 | 0.615 | 0.0188 | Yes |
| 6 | RPS26 |  |  | 9 | 0.595 | 0.0225 | Yes |
| 7 | RPL36A |  |  | 10 | 0.588 | 0.0261 | Yes |
| 8 | RPL27 |  |  | 11 | 0.587 | 0.0298 | Yes |
| 9 | RPL12 |  |  | 12 | 0.579 | 0.0333 | Yes |
| 10 | UQCRQ |  |  | 13 | 0.574 | 0.0369 | Yes |
| 11 | RPL6 |  |  | 16 | 0.555 | 0.0398 | Yes |
| 12 | RPL35 |  |  | 17 | 0.549 | 0.0432 | Yes |
| 13 | RPS25 |  |  | 19 | 0.527 | 0.0462 | Yes |
| 14 | RPL37 |  |  | 20 | 0.526 | 0.0495 | Yes |
| 15 | RPL24 |  |  | 21 | 0.524 | 0.0527 | Yes |
| 16 | RPL22 |  |  | 22 | 0.520 | 0.0559 | Yes |
| 17 | RPLP1 |  |  | 25 | 0.514 | 0.0586 | Yes |
| 18 | SH3BGRL3 |  |  | 26 | 0.505 | 0.0617 | Yes |
| 19 | RPL23A |  |  | 27 | 0.502 | 0.0648 | Yes |
| 20 | EEF1B2 |  |  | 28 | 0.501 | 0.0679 | Yes |
| 21 | SNRPD2 |  |  | 29 | 0.500 | 0.0710 | Yes |
| 22 | RPL28 |  |  | 32 | 0.498 | 0.0736 | Yes |
| 23 | UBA52 |  |  | 33 | 0.494 | 0.0766 | Yes |
| 24 | RPS13 |  |  | 35 | 0.490 | 0.0794 | Yes |
| 25 | RPLP2 |  |  | 36 | 0.489 | 0.0824 | Yes |
| 26 | POLR2L |  |  | 38 | 0.482 | 0.0851 | Yes |
| 27 | RPS21 |  |  | 39 | 0.482 | 0.0881 | Yes |
| 28 | RPS6 |  |  | 41 | 0.480 | 0.0908 | Yes |
| 29 | RPL14 |  |  | 42 | 0.479 | 0.0938 | Yes |
| 30 | RPL8 |  |  | 44 | 0.469 | 0.0964 | Yes |
| 31 | RPL13A |  |  | 45 | 0.467 | 0.0993 | Yes |
| 32 | TXN |  |  | 47 | 0.465 | 0.1019 | Yes |
| 33 | S100A11 |  |  | 48 | 0.464 | 0.1048 | Yes |
| 34 | RPS20 |  |  | 49 | 0.463 | 0.1077 | Yes |
| 35 | RPL5 |  |  | 51 | 0.461 | 0.1103 | Yes |
| 36 | NDUFB2 |  |  | 52 | 0.460 | 0.1131 | Yes |
| 37 | RPL32 |  |  | 55 | 0.457 | 0.1154 | Yes |
| 38 | RPS5 |  |  | 57 | 0.454 | 0.1179 | Yes |
| 39 | RPL18A |  |  | 58 | 0.453 | 0.1208 | Yes |
| 40 | CYCS |  |  | 59 | 0.453 | 0.1236 | Yes |
| 41 | RPS10 |  |  | 60 | 0.452 | 0.1264 | Yes |
| 42 | RPS18 |  |  | 61 | 0.451 | 0.1292 | Yes |
| 43 | RPL36 |  |  | 62 | 0.449 | 0.1320 | Yes |
| 44 | COX8A |  |  | 63 | 0.448 | 0.1348 | Yes |
| 45 | RPL37A |  |  | 64 | 0.448 | 0.1375 | Yes |
| 46 | ZFAS1 |  |  | 65 | 0.447 | 0.1403 | Yes |
| 47 | RPL26 |  |  | 66 | 0.447 | 0.1431 | Yes |
| 48 | RPS24 |  |  | 69 | 0.444 | 0.1453 | Yes |
| 49 | RPL30 |  |  | 71 | 0.442 | 0.1477 | Yes |
| 50 | RPS28 |  |  | 73 | 0.440 | 0.1502 | Yes |
| 51 | RPS7 |  |  | 75 | 0.436 | 0.1526 | Yes |
| 52 | RPL11 |  |  | 76 | 0.436 | 0.1553 | Yes |
| 53 | RPS3A |  |  | 82 | 0.429 | 0.1566 | Yes |
| 54 | OST4 |  |  | 83 | 0.429 | 0.1593 | Yes |
| 55 | RPS19 |  |  | 84 | 0.427 | 0.1619 | Yes |
| 56 | RPS3 |  |  | 90 | 0.420 | 0.1631 | Yes |
| 57 | RPS27A |  |  | 95 | 0.413 | 0.1645 | Yes |
| 58 | RPL35A |  |  | 96 | 0.413 | 0.1671 | Yes |
| 59 | VDAC1 |  |  | 98 | 0.413 | 0.1694 | Yes |
| 60 | S100A10 |  |  | 102 | 0.409 | 0.1711 | Yes |
| 61 | RPL27A |  |  | 104 | 0.408 | 0.1733 | Yes |
| 62 | RPL34 |  |  | 105 | 0.407 | 0.1759 | Yes |
| 63 | RPSA |  |  | 108 | 0.405 | 0.1778 | Yes |
| 64 | SERF2 |  |  | 111 | 0.403 | 0.1798 | Yes |
| 65 | FAU |  |  | 113 | 0.400 | 0.1820 | Yes |
| 66 | CALM2 |  |  | 120 | 0.396 | 0.1827 | Yes |
| 67 | PPIA |  |  | 121 | 0.395 | 0.1852 | Yes |
| 68 | SNRPB |  |  | 122 | 0.395 | 0.1876 | Yes |
| 69 | PABPC1 |  |  | 123 | 0.394 | 0.1901 | Yes |
| 70 | NOL7 |  |  | 126 | 0.392 | 0.1920 | Yes |
| 71 | RPS23 |  |  | 129 | 0.391 | 0.1938 | Yes |
| 72 | RPS27 |  |  | 132 | 0.388 | 0.1957 | Yes |
| 73 | NDUFA4 |  |  | 140 | 0.382 | 0.1961 | Yes |
| 74 | RPL21 |  |  | 141 | 0.382 | 0.1984 | Yes |
| 75 | RPL36AL |  |  | 144 | 0.381 | 0.2002 | Yes |
| 76 | COX6A1 |  |  | 145 | 0.379 | 0.2026 | Yes |
| 77 | TMSB10 |  |  | 148 | 0.376 | 0.2044 | Yes |
| 78 | RPL13 |  |  | 152 | 0.372 | 0.2058 | Yes |
| 79 | ACTG1 |  |  | 153 | 0.371 | 0.2081 | Yes |
| 80 | PPDPF |  |  | 157 | 0.369 | 0.2096 | Yes |
| 81 | FKBP1A |  |  | 158 | 0.368 | 0.2119 | Yes |
| 82 | MRPL17 |  |  | 159 | 0.368 | 0.2142 | Yes |
| 83 | MYL12B |  |  | 164 | 0.365 | 0.2153 | Yes |
| 84 | YBX1 |  |  | 167 | 0.364 | 0.2170 | Yes |
| 85 | COX7C |  |  | 170 | 0.362 | 0.2187 | Yes |
| 86 | COMMD6 |  |  | 173 | 0.359 | 0.2203 | Yes |
| 87 | RPL18 |  |  | 178 | 0.355 | 0.2214 | Yes |
| 88 | RPS15 |  |  | 180 | 0.353 | 0.2233 | Yes |
| 89 | FTH1 |  |  | 181 | 0.352 | 0.2255 | Yes |
| 90 | UBL5 |  |  | 182 | 0.352 | 0.2277 | Yes |
| 91 | SUGT1 |  |  | 185 | 0.349 | 0.2293 | Yes |
| 92 | SNRPG |  |  | 186 | 0.349 | 0.2315 | Yes |
| 93 | RPS27L |  |  | 188 | 0.348 | 0.2333 | Yes |
| 94 | CHCHD2 |  |  | 195 | 0.341 | 0.2338 | Yes |
| 95 | COTL1 |  |  | 198 | 0.340 | 0.2353 | Yes |
| 96 | RPL29 |  |  | 201 | 0.338 | 0.2369 | Yes |
| 97 | EIF5B |  |  | 204 | 0.336 | 0.2384 | Yes |
| 98 | HINT1 |  |  | 205 | 0.335 | 0.2405 | Yes |
| 99 | CALM1 |  |  | 209 | 0.334 | 0.2417 | Yes |
| 100 | MRPL20 |  |  | 213 | 0.331 | 0.2429 | Yes |
| 101 | RPS9 |  |  | 214 | 0.331 | 0.2450 | Yes |
| 102 | UBE2L3 |  |  | 216 | 0.331 | 0.2467 | Yes |
| 103 | RANBP1 |  |  | 221 | 0.328 | 0.2476 | Yes |
| 104 | PTMA |  |  | 222 | 0.327 | 0.2497 | Yes |
| 105 | ST13 |  |  | 227 | 0.325 | 0.2506 | Yes |
| 106 | RPS2 |  |  | 228 | 0.325 | 0.2526 | Yes |
| 107 | ATOX1 |  |  | 233 | 0.321 | 0.2534 | Yes |
| 108 | UQCC2 |  |  | 234 | 0.320 | 0.2554 | Yes |
| 109 | BUD31 |  |  | 237 | 0.316 | 0.2568 | Yes |
| 110 | SAP18 |  |  | 238 | 0.315 | 0.2588 | Yes |
| 111 | ENO1 |  |  | 241 | 0.314 | 0.2602 | Yes |
| 112 | POMP |  |  | 242 | 0.313 | 0.2621 | Yes |
| 113 | RPS15A |  |  | 245 | 0.312 | 0.2635 | Yes |
| 114 | NIFK |  |  | 246 | 0.312 | 0.2654 | Yes |
| 115 | SRP14 |  |  | 247 | 0.311 | 0.2674 | Yes |
| 116 | MRPS18C |  |  | 251 | 0.310 | 0.2684 | Yes |
| 117 | RPL7A |  |  | 255 | 0.308 | 0.2695 | Yes |
| 118 | RPS16 |  |  | 256 | 0.308 | 0.2714 | Yes |
| 119 | TOMM7 |  |  | 264 | 0.303 | 0.2713 | Yes |
| 120 | RPL41 |  |  | 265 | 0.303 | 0.2732 | Yes |
| 121 | LSM4 |  |  | 268 | 0.303 | 0.2745 | Yes |
| 122 | RPL9 |  |  | 269 | 0.302 | 0.2764 | Yes |
| 123 | TMSB4X |  |  | 273 | 0.300 | 0.2774 | Yes |
| 124 | RBM8A |  |  | 276 | 0.298 | 0.2787 | Yes |
| 125 | NDUFC1 |  |  | 284 | 0.296 | 0.2786 | Yes |
| 126 | RPL10 |  |  | 285 | 0.296 | 0.2804 | Yes |
| 127 | MYL9 |  |  | 293 | 0.293 | 0.2803 | Yes |
| 128 | YWHAH |  |  | 297 | 0.291 | 0.2812 | Yes |
| 129 | SRP9 |  |  | 309 | 0.289 | 0.2799 | Yes |
| 130 | LLPH |  |  | 313 | 0.288 | 0.2809 | Yes |
| 131 | BANF1 |  |  | 317 | 0.287 | 0.2818 | Yes |
| 132 | GAPDH |  |  | 320 | 0.286 | 0.2830 | Yes |
| 133 | MINOS1 |  |  | 321 | 0.286 | 0.2848 | Yes |
| 134 | MRPL36 |  |  | 327 | 0.285 | 0.2852 | Yes |
| 135 | MRPS5 |  |  | 342 | 0.280 | 0.2830 | Yes |
| 136 | BTF3 |  |  | 344 | 0.279 | 0.2844 | Yes |
| 137 | ZC3H15 |  |  | 347 | 0.278 | 0.2856 | Yes |
| 138 | NAA38 |  |  | 354 | 0.274 | 0.2856 | Yes |
| 139 | DNAJA1 |  |  | 355 | 0.273 | 0.2873 | Yes |
| 140 | RPS11 |  |  | 358 | 0.272 | 0.2884 | Yes |
| 141 | RPL15 |  |  | 363 | 0.271 | 0.2890 | Yes |
| 142 | RBM17 |  |  | 364 | 0.271 | 0.2906 | Yes |
| 143 | C11orf58 |  |  | 365 | 0.271 | 0.2923 | Yes |
| 144 | SARS |  |  | 367 | 0.270 | 0.2937 | Yes |
| 145 | NDUFA11 |  |  | 371 | 0.269 | 0.2945 | Yes |
| 146 | MTIF3 |  |  | 374 | 0.269 | 0.2957 | Yes |
| 147 | SNRPD1 |  |  | 376 | 0.268 | 0.2970 | Yes |
| 148 | TOP1 |  |  | 382 | 0.267 | 0.2973 | Yes |
| 149 | H2AFZ |  |  | 386 | 0.265 | 0.2981 | Yes |
| 150 | CCDC90B |  |  | 390 | 0.263 | 0.2989 | Yes |
| 151 | RPL7 |  |  | 394 | 0.262 | 0.2997 | Yes |
| 152 | VPS29 |  |  | 402 | 0.259 | 0.2993 | Yes |
| 153 | TAGLN |  |  | 407 | 0.257 | 0.2998 | Yes |
| 154 | FAM204A |  |  | 415 | 0.255 | 0.2994 | Yes |
| 155 | CWC15 |  |  | 422 | 0.253 | 0.2993 | Yes |
| 156 | NDUFB4 |  |  | 427 | 0.252 | 0.2997 | Yes |
| 157 | UQCRH |  |  | 429 | 0.250 | 0.3010 | Yes |
| 158 | ALDOA |  |  | 440 | -0.251 | 0.2997 | No |
| 159 | FILIP1L |  |  | 454 | -0.252 | 0.2976 | No |
| 160 | PPFIBP1 |  |  | 462 | -0.252 | 0.2972 | No |
| 161 | MYLK |  |  | 479 | -0.253 | 0.2943 | No |
| 162 | SACS |  |  | 510 | -0.255 | 0.2874 | No |
| 163 | ST3GAL5 |  |  | 519 | -0.255 | 0.2867 | No |
| 164 | ACSL3 |  |  | 529 | -0.256 | 0.2858 | No |
| 165 | HIPK2 |  |  | 546 | -0.257 | 0.2829 | No |
| 166 | PTPRA |  |  | 552 | -0.257 | 0.2831 | No |
| 167 | TLE1 |  |  | 555 | -0.257 | 0.2841 | No |
| 168 | PHIP |  |  | 560 | -0.257 | 0.2846 | No |
| 169 | CHD9 |  |  | 574 | -0.258 | 0.2825 | No |
| 170 | PHLDA1 |  |  | 589 | -0.259 | 0.2802 | No |
| 171 | DHCR24 |  |  | 598 | -0.260 | 0.2795 | No |
| 172 | MLEC |  |  | 627 | -0.261 | 0.2733 | No |
| 173 | ARGLU1 |  |  | 633 | -0.261 | 0.2735 | No |
| 174 | TM9SF2 |  |  | 664 | -0.263 | 0.2667 | No |
| 175 | REV3L |  |  | 693 | -0.264 | 0.2604 | No |
| 176 | PRDX2 |  |  | 708 | -0.265 | 0.2581 | No |
| 177 | ARID4B |  |  | 742 | -0.267 | 0.2505 | No |
| 178 | IGF2BP3 |  |  | 754 | -0.267 | 0.2490 | No |
| 179 | UNC50 |  |  | 763 | -0.267 | 0.2485 | No |
| 180 | KMT2A |  |  | 773 | -0.268 | 0.2476 | No |
| 181 | ARL6IP5 |  |  | 775 | -0.268 | 0.2490 | No |
| 182 | CHD1 |  |  | 812 | -0.270 | 0.2405 | No |
| 183 | FRMD4A |  |  | 819 | -0.270 | 0.2405 | No |
| 184 | ID3 |  |  | 880 | -0.273 | 0.2253 | No |
| 185 | VEZT |  |  | 909 | -0.275 | 0.2191 | No |
| 186 | BPTF |  |  | 925 | -0.276 | 0.2166 | No |
| 187 | ATP1B3 |  |  | 937 | -0.276 | 0.2152 | No |
| 188 | ASAP1 |  |  | 1002 | -0.280 | 0.1989 | No |
| 189 | MCM3 |  |  | 1021 | -0.280 | 0.1956 | No |
| 190 | PMP22 |  |  | 1023 | -0.281 | 0.1971 | No |
| 191 | MAP1A |  |  | 1046 | -0.282 | 0.1926 | No |
| 192 | THOC6 |  |  | 1063 | -0.283 | 0.1899 | No |
| 193 | WWTR1 |  |  | 1080 | -0.284 | 0.1871 | No |
| 194 | PCNA |  |  | 1116 | -0.286 | 0.1790 | No |
| 195 | MAGEF1 |  |  | 1172 | -0.288 | 0.1653 | No |
| 196 | TMEM165 |  |  | 1201 | -0.290 | 0.1593 | No |
| 197 | GOLGB1 |  |  | 1232 | -0.292 | 0.1526 | No |
| 198 | ARSJ |  |  | 1233 | -0.292 | 0.1544 | No |
| 199 | ASH1L |  |  | 1244 | -0.292 | 0.1534 | No |
| 200 | RMI2 |  |  | 1249 | -0.292 | 0.1541 | No |
| 201 | LRRC58 |  |  | 1250 | -0.292 | 0.1559 | No |
| 202 | RUFY3 |  |  | 1257 | -0.293 | 0.1561 | No |
| 203 | NUSAP1 |  |  | 1262 | -0.293 | 0.1568 | No |
| 204 | SLC4A7 |  |  | 1314 | -0.295 | 0.1442 | No |
| 205 | ELOVL6 |  |  | 1332 | -0.296 | 0.1413 | No |
| 206 | NR2F1 |  |  | 1340 | -0.297 | 0.1411 | No |
| 207 | BTG2 |  |  | 1350 | -0.297 | 0.1405 | No |
| 208 | TGIF1 |  |  | 1506 | -0.306 | 0.0987 | No |
| 209 | RNF13 |  |  | 1521 | -0.307 | 0.0967 | No |
| 210 | SEC63 |  |  | 1534 | -0.308 | 0.0952 | No |
| 211 | NKTR |  |  | 1552 | -0.309 | 0.0923 | No |
| 212 | INPP5F |  |  | 1558 | -0.310 | 0.0929 | No |
| 213 | SPRY2 |  |  | 1592 | -0.311 | 0.0855 | No |
| 214 | KLF6 |  |  | 1596 | -0.312 | 0.0866 | No |
| 215 | ARHGAP29 |  |  | 1614 | -0.313 | 0.0837 | No |
| 216 | CDKN1A |  |  | 1631 | -0.314 | 0.0812 | No |
| 217 | RNF149 |  |  | 1644 | -0.315 | 0.0798 | No |
| 218 | HOXB3 |  |  | 1700 | -0.318 | 0.0663 | No |
| 219 | DUSP4 |  |  | 1703 | -0.319 | 0.0677 | No |
| 220 | CLCN3 |  |  | 1777 | -0.323 | 0.0491 | No |
| 221 | ID2 |  |  | 1780 | -0.323 | 0.0506 | No |
| 222 | RBBP6 |  |  | 1782 | -0.324 | 0.0523 | No |
| 223 | JMJD1C |  |  | 1823 | -0.325 | 0.0431 | No |
| 224 | GLG1 |  |  | 1839 | -0.326 | 0.0409 | No |
| 225 | PXK |  |  | 1871 | -0.328 | 0.0342 | No |
| 226 | ANKRD11 |  |  | 1878 | -0.328 | 0.0345 | No |
| 227 | PTPRE |  |  | 1880 | -0.328 | 0.0363 | No |
| 228 | CXXC5 |  |  | 1889 | -0.329 | 0.0361 | No |
| 229 | DAAM1 |  |  | 1915 | -0.330 | 0.0311 | No |
| 230 | TUBA1A |  |  | 1916 | -0.330 | 0.0331 | No |
| 231 | SESN3 |  |  | 1920 | -0.331 | 0.0343 | No |
| 232 | UBE2C |  |  | 1942 | -0.332 | 0.0305 | No |
| 233 | EFHC1 |  |  | 1946 | -0.332 | 0.0317 | No |
| 234 | DDX17 |  |  | 1950 | -0.332 | 0.0329 | No |
| 235 | SLC2A1 |  |  | 1961 | -0.333 | 0.0322 | No |
| 236 | SALL1 |  |  | 1962 | -0.333 | 0.0342 | No |
| 237 | SMC2 |  |  | 1963 | -0.333 | 0.0363 | No |
| 238 | LBR |  |  | 1973 | -0.334 | 0.0358 | No |
| 239 | ZNF292 |  |  | 1992 | -0.335 | 0.0329 | No |
| 240 | LYPD1 |  |  | 2023 | -0.337 | 0.0265 | No |
| 241 | DDIT4 |  |  | 2027 | -0.337 | 0.0277 | No |
| 242 | GTF2I |  |  | 2067 | -0.339 | 0.0189 | No |
| 243 | MTDH |  |  | 2083 | -0.340 | 0.0168 | No |
| 244 | XBP1 |  |  | 2097 | -0.341 | 0.0152 | No |
| 245 | TRIB2 |  |  | 2105 | -0.342 | 0.0154 | No |
| 246 | FAM20C |  |  | 2143 | -0.345 | 0.0071 | No |
| 247 | TGOLN2 |  |  | 2235 | -0.351 | -0.0164 | No |
| 248 | INSIG1 |  |  | 2266 | -0.354 | -0.0226 | No |
| 249 | TPX2 |  |  | 2289 | -0.356 | -0.0266 | No |
| 250 | ABL2 |  |  | 2311 | -0.358 | -0.0303 | No |
| 251 | BSG |  |  | 2316 | -0.359 | -0.0292 | No |
| 252 | DLG1 |  |  | 2317 | -0.359 | -0.0270 | No |
| 253 | CCNDBP1 |  |  | 2319 | -0.359 | -0.0250 | No |
| 254 | PDLIM4 |  |  | 2346 | -0.361 | -0.0301 | No |
| 255 | TCF12 |  |  | 2360 | -0.362 | -0.0315 | No |
| 256 | SPECC1 |  |  | 2368 | -0.363 | -0.0312 | No |
| 257 | IQGAP1 |  |  | 2405 | -0.366 | -0.0391 | No |
| 258 | DLGAP5 |  |  | 2421 | -0.367 | -0.0410 | No |
| 259 | SLITRK6 |  |  | 2424 | -0.367 | -0.0393 | No |
| 260 | ZKSCAN1 |  |  | 2437 | -0.368 | -0.0404 | No |
| 261 | U2AF1 |  |  | 2460 | -0.370 | -0.0443 | No |
| 262 | PRCP |  |  | 2489 | -0.372 | -0.0499 | No |
| 263 | PDIA6 |  |  | 2505 | -0.373 | -0.0518 | No |
| 264 | VGLL4 |  |  | 2525 | -0.375 | -0.0548 | No |
| 265 | SDC2 |  |  | 2537 | -0.376 | -0.0556 | No |
| 266 | ROBO1 |  |  | 2546 | -0.377 | -0.0555 | No |
| 267 | FLNA |  |  | 2559 | -0.378 | -0.0565 | No |
| 268 | BAZ2B |  |  | 2576 | -0.380 | -0.0586 | No |
| 269 | DYNC1H1 |  |  | 2580 | -0.380 | -0.0571 | No |
| 270 | FADS1 |  |  | 2588 | -0.381 | -0.0567 | No |
| 271 | SOX4 |  |  | 2595 | -0.381 | -0.0561 | No |
| 272 | PNISR |  |  | 2607 | -0.383 | -0.0568 | No |
| 273 | TACC2 |  |  | 2615 | -0.383 | -0.0564 | No |
| 274 | MLLT3 |  |  | 2633 | -0.384 | -0.0588 | No |
| 275 | HSD11B1L |  |  | 2641 | -0.385 | -0.0584 | No |
| 276 | SPTBN1 |  |  | 2647 | -0.385 | -0.0574 | No |
| 277 | MACF1 |  |  | 2670 | -0.388 | -0.0612 | No |
| 278 | HMGCR |  |  | 2673 | -0.388 | -0.0593 | No |
| 279 | KIF20B |  |  | 2689 | -0.390 | -0.0611 | No |
| 280 | HOXB5 |  |  | 2713 | -0.392 | -0.0652 | No |
| 281 | TACC1 |  |  | 2728 | -0.393 | -0.0667 | No |
| 282 | ANKRD1 |  |  | 2755 | -0.395 | -0.0715 | No |
| 283 | PRR7 |  |  | 2779 | -0.397 | -0.0755 | No |
| 284 | TNIK |  |  | 2809 | -0.400 | -0.0812 | No |
| 285 | FHL2 |  |  | 2851 | -0.403 | -0.0903 | No |
| 286 | UBTD1 |  |  | 2852 | -0.403 | -0.0878 | No |
| 287 | SMC4 |  |  | 2872 | -0.405 | -0.0906 | No |
| 288 | SVIL |  |  | 2912 | -0.409 | -0.0990 | No |
| 289 | NINJ1 |  |  | 2923 | -0.411 | -0.0993 | No |
| 290 | JUN |  |  | 2935 | -0.413 | -0.0998 | No |
| 291 | CD81 |  |  | 2945 | -0.414 | -0.0998 | No |
| 292 | RHOBTB3 |  |  | 2955 | -0.415 | -0.0998 | No |
| 293 | CSRP2 |  |  | 2974 | -0.417 | -0.1022 | No |
| 294 | ARL4C |  |  | 2985 | -0.418 | -0.1025 | No |
| 295 | IRX3 |  |  | 3001 | -0.420 | -0.1041 | No |
| 296 | ITGB8 |  |  | 3026 | -0.424 | -0.1082 | No |
| 297 | NFIB |  |  | 3102 | -0.431 | -0.1267 | No |
| 298 | AKAP9 |  |  | 3154 | -0.438 | -0.1383 | No |
| 299 | ENC1 |  |  | 3169 | -0.440 | -0.1395 | No |
| 300 | RTN4 |  |  | 3179 | -0.441 | -0.1393 | No |
| 301 | AHNAK2 |  |  | 3190 | -0.442 | -0.1394 | No |
| 302 | HSPA5 |  |  | 3193 | -0.442 | -0.1372 | No |
| 303 | MYO10 |  |  | 3196 | -0.442 | -0.1350 | No |
| 304 | SYT1 |  |  | 3200 | -0.442 | -0.1331 | No |
| 305 | WSB1 |  |  | 3218 | -0.445 | -0.1351 | No |
| 306 | MSMO1 |  |  | 3241 | -0.450 | -0.1385 | No |
| 307 | PTPRG |  |  | 3246 | -0.451 | -0.1369 | No |
| 308 | TRAM2 |  |  | 3247 | -0.451 | -0.1341 | No |
| 309 | GATA3 |  |  | 3260 | -0.453 | -0.1346 | No |
| 310 | BMP2 |  |  | 3307 | -0.462 | -0.1447 | No |
| 311 | PLEC |  |  | 3324 | -0.466 | -0.1463 | No |
| 312 | SLC20A1 |  |  | 3344 | -0.470 | -0.1488 | No |
| 313 | MCAM |  |  | 3353 | -0.472 | -0.1481 | No |
| 314 | LINC00511 |  |  | 3379 | -0.475 | -0.1522 | No |
| 315 | RAB11FIP1 |  |  | 3394 | -0.480 | -0.1531 | No |
| 316 | PLAU |  |  | 3435 | -0.488 | -0.1614 | No |
| 317 | TPST2 |  |  | 3437 | -0.489 | -0.1586 | No |
| 318 | RNF180 |  |  | 3455 | -0.494 | -0.1603 | No |
| 319 | ZFP36L1 |  |  | 3487 | -0.503 | -0.1659 | No |
| 320 | UGCG |  |  | 3532 | -0.521 | -0.1751 | No |
| 321 | DCBLD2 |  |  | 3543 | -0.525 | -0.1747 | No |
| 322 | CCDC88A |  |  | 3549 | -0.526 | -0.1728 | No |
| 323 | PDP1 |  |  | 3555 | -0.528 | -0.1709 | No |
| 324 | DKK1 |  |  | 3556 | -0.530 | -0.1676 | No |
| 325 | SOX9 |  |  | 3560 | -0.531 | -0.1652 | No |
| 326 | CKB |  |  | 3572 | -0.536 | -0.1649 | No |
| 327 | ATP2B1 |  |  | 3580 | -0.539 | -0.1636 | No |
| 328 | TUBB2B |  |  | 3594 | -0.544 | -0.1639 | No |
| 329 | RRBP1 |  |  | 3595 | -0.545 | -0.1605 | No |
| 330 | NES |  |  | 3596 | -0.545 | -0.1571 | No |
| 331 | DST |  |  | 3597 | -0.545 | -0.1537 | No |
| 332 | VCL |  |  | 3603 | -0.551 | -0.1517 | No |
| 333 | SPRY1 |  |  | 3605 | -0.552 | -0.1485 | No |
| 334 | SQLE |  |  | 3606 | -0.553 | -0.1451 | No |
| 335 | SOX2 |  |  | 3613 | -0.555 | -0.1434 | No |
| 336 | RGS16 |  |  | 3616 | -0.555 | -0.1405 | No |
| 337 | FOS |  |  | 3621 | -0.561 | -0.1381 | No |
| 338 | ANLN |  |  | 3625 | -0.562 | -0.1355 | No |
| 339 | CD9 |  |  | 3634 | -0.567 | -0.1342 | No |
| 340 | TPBG |  |  | 3638 | -0.570 | -0.1315 | No |
| 341 | DUSP6 |  |  | 3648 | -0.577 | -0.1305 | No |
| 342 | PRSS23 |  |  | 3658 | -0.584 | -0.1294 | No |
| 343 | AHNAK |  |  | 3660 | -0.584 | -0.1260 | No |
| 344 | CXADR |  |  | 3670 | -0.591 | -0.1249 | No |
| 345 | POLR2J3 |  |  | 3689 | -0.608 | -0.1262 | No |
| 346 | TCF4 |  |  | 3696 | -0.612 | -0.1241 | No |
| 347 | CDK6 |  |  | 3700 | -0.614 | -0.1211 | No |
| 348 | ZBTB20 |  |  | 3702 | -0.617 | -0.1176 | No |
| 349 | LMO4 |  |  | 3734 | -0.650 | -0.1222 | No |
| 350 | PMEPA1 |  |  | 3737 | -0.657 | -0.1187 | No |
| 351 | AKAP12 |  |  | 3740 | -0.660 | -0.1152 | No |
| 352 | TIMP3 |  |  | 3755 | -0.677 | -0.1149 | No |
| 353 | ARL6IP1 |  |  | 3759 | -0.685 | -0.1115 | No |
| 354 | FZD3 |  |  | 3765 | -0.695 | -0.1086 | No |
| 355 | TMEFF2 |  |  | 3767 | -0.697 | -0.1046 | No |
| 356 | LIMA1 |  |  | 3774 | -0.706 | -0.1019 | No |
| 357 | SCG2 |  |  | 3786 | -0.718 | -0.1005 | No |
| 358 | MKI67 |  |  | 3788 | -0.721 | -0.0963 | No |
| 359 | CELF2 |  |  | 3822 | -0.774 | -0.1008 | No |
| 360 | BEX1 |  |  | 3825 | -0.779 | -0.0965 | No |
| 361 | MIAT |  |  | 3833 | -0.794 | -0.0936 | No |
| 362 | EDN1 |  |  | 3838 | -0.812 | -0.0897 | No |
| 363 | CENPF |  |  | 3842 | -0.820 | -0.0854 | No |
| 364 | CADM1 |  |  | 3856 | -0.851 | -0.0838 | No |
| 365 | LRRN3 |  |  | 3859 | -0.856 | -0.0790 | No |
| 366 | MEIS2 |  |  | 3862 | -0.870 | -0.0742 | No |
| 367 | KCNQ1OT1 |  |  | 3866 | -0.885 | -0.0695 | No |
| 368 | FABP7 |  |  | 3867 | -0.888 | -0.0640 | No |
| 369 | LINC00461 |  |  | 3870 | -0.925 | -0.0588 | No |
| 370 | TNC |  |  | 3874 | -0.951 | -0.0538 | No |
| 371 | POU3F2 |  |  | 3875 | -0.953 | -0.0479 | No |
| 372 | CYR61 |  |  | 3887 | -1.037 | -0.0445 | No |
| 373 | GPM6B |  |  | 3889 | -1.047 | -0.0383 | No |
| 374 | IGFBP5 |  |  | 3895 | -1.082 | -0.0330 | No |
| 375 | ASPM |  |  | 3899 | -1.166 | -0.0266 | No |
| 376 | CTGF |  |  | 3901 | -1.185 | -0.0195 | No |
| 377 | PTPRZ1 |  |  | 3913 | -1.342 | -0.0143 | No |
| 378 | HIST1H4C |  |  | 3914 | -1.352 | -0.0059 | No |
| 379 | MALAT1 |  |  | 3924 | -1.583 | 0.0014 | No |
Table: GSEA details [plain text format]

  

Fig 2: FELDMAN\_2020\_LOWRNA: Random ES distribution      
 Gene set null distribution of ES for **FELDMAN\_2020\_LOWRNA**
